# Clinical factors predicting tic persistence into adulthood

**DOI:** 10.3389/fpsyt.2026.1948693

**Published:** 2026-10-06

**Authors:** Ariadne Kaylor, Joshua A. Senior, Evan Realbuto, Jenna LaRochelle, Dongmei Yu, Daisy T. Noriega-Makarskyy, Angela Essa, Carol A. Mathews, Jeremiah M. Scharf

**Affiliations:** 1 Psychiatric and Neurodevelopmental Genetics Unit, Center for Genomic Medicine, Massachusetts General Hospital, Boston, MA, United States; 2 Stanley Center for Psychiatric Research, Broad Institute of MIT and Harvard, Cambridge, MA, United States; 3 Department of Psychiatry, University of Florida, Gainesville, FL, United States; 4 Genetics Institute, University of Florida, Gainesville, FL, United States; 5 Center for OCD, Anxiety and Related Disorders, McKnight Brain Institute, University of Florida, Gainesville, FL, United States; 6 Department of Psychiatry, Massachusetts General Hospital, Harvard Medical School, Boston, MA, United States; 7 Department of Neurology, Brigham and Women’s Hospital, Harvard Medical School, Boston, MA, United States; 8 Department of Neurology, Massachusetts General Hospital, Harvard Medical School, Boston, MA, United States

**Keywords:** significant life events, stress, tic disorders, tic severity, Tourette syndrome

## Abstract

**Background:**

Despite widespread patient and family interest in long-term outcome prediction at the time of Tourette syndrome (TS) diagnosis, there is limited data on factors associated with disease severity and impairment in adulthood. This study aimed to examine the relationship between childhood and adulthood worst-ever tics and identify predictors of adulthood tic severity and impairment, including the role of significant life events.

**Methods:**

In a cross-sectional design, 258 adult participants with TS completed an online questionnaire assessing tic severity and impairment during their worst-ever tic periods in childhood and adulthood, co-occurring obsessive-compulsive disorder (OCD) and attention deficit hyperactivity disorder (ADHD) symptoms and clinical and demographic characteristics.

**Results:**

Worst-ever childhood tic severity and impairment were significantly associated with greater worst-ever adulthood tic severity and impairment. Co-occurring OCD was also associated with greater tic severity and impairment in adulthood. Use of tic medication was a significant predictor of greater worst-ever adulthood tic impairment, but not severity. Overall, 153 (59.3%) participants reported experiencing a significant life event at the time of their worst-ever tics in adulthood, and the presence of a significant life event was associated with greater worst-ever adulthood tic impairment. There was a significant effect of negative and neutral life events, but not positive life events, on adult worst-ever tic severity and impairment, compared to those not reporting a significant life event.

**Discussion:**

These findings provide insight into characteristics present either in childhood or in adulthood that may help predict the presence and severity of tic symptoms in adulthood.

## 1 Introduction

Tourette syndrome (TS) is a childhood-onset neurodevelopmental disorder that requires the presence of multiple involuntary, repetitive motor movements and at least one vocalization (tics) that wax and wane for at least a year (1). While TS has a strong neurobiological basis, environmental factors clearly play a role in disease development and in tic exacerbation. Additional factors—in particular, the presence of co-occurring psychiatric disorders—also contribute to tic exacerbation (2, 3).

Tics typically begin between ages 4 and 6 as simple motor tics, followed by the emergence of vocal tics and more complex motor tics (4). Although tic trajectories vary substantially, many individuals with TS experience their worst tic severity in late childhood or early adolescence, followed by symptom improvement during adolescence and into adulthood (5–7). By adulthood, tic symptoms tend to improve and become mild in nature, although it is unclear how many individuals with TS experience a complete remission from tics (8). In a large prospective clinical study of 314 participants at baseline and 227 participants at 6-year follow-up, Groth et al. found that among participants older than 16 years, 17.7% had no tics, 59.5% had minimal or mild tics, and 22.8% continued to have moderate or severe tics, with tic severity declining by 0.8 YGTSS points per year during adolescence (6).

Tic-related impairment also tends to lessen or remit in adulthood. In a survey of 83 adults with TS, Byler et al. found that 62 of 79 respondents (78.5%) reported that their tics either did not affect everyday life or did not require adjustment despite some impact (9). A separate study that included a 25 to 32-year follow-up in 45 individuals with TS found that most reported tic improvement over time and generally had favorable psychosocial outcomes, including academic attainment, employment, marriage, and high subjective quality of life (10).

In children and adolescents, short-term fluctuations in tic severity are not fully understood, but available evidence suggests that tic expression is associated with complex interactions between major life events, daily stressors, underlying psychological or psychiatric symptoms, and treatment choices, both of tics and of co-occurring psychiatric disorders (3). Short-term tic severity may also be modified by behavioral or pharmacologic treatment, although the effect of treatment in childhood on long-term tic persistence into adulthood remains unclear (11). Although potentially reducing tic severity and impairment, available evidence does not yet establish whether treatment for tics or co-occurring psychiatric conditions in childhood alters the long-term trajectory of tic persistence into adulthood (11, 12).

Although the causes of tic persistence, and in particular, tic severity, in adulthood are not well known, several predictors have been identified. In the study by Groth et al., childhood tic severity was the strongest predictor of high tic severity at 6-year follow-up, with each 1-point increase in childhood tic severity associated with a 9% increase in the odds of a higher tic severity score in later adolescence or young adulthood (13). In this study, family history of highly comorbid TS, defined as TS with co-occurring OCD and ADHD, was also associated with higher tic severity at follow-up (13). Similarly, in an 11-year follow-up study of 80 adolescents and adults with TS, Ricketts et al. found that higher parent-reported tic severity in childhood was associated with greater tic severity and tic-related impairment in adulthood (14). Mataix-Cols et al. also found that tic disorder persistence into adulthood was associated with childhood psychiatric comorbidities and family history of psychiatric disorders, particularly family history of tic and anxiety disorders (15). In particular, ADHD, OCD, pervasive developmental disorders, and anxiety disorders in childhood were highly correlated with tic persistence into adulthood (15).

Furthermore, Lin et al. reported in a 2-year prospective study of children and adolescents with TS and/or OCD that baseline depressive symptoms were independent predictors of future tic severity (16). Additional reported potential predictors of higher tic severity or impairment in adulthood include female sex, higher reported tic severity in childhood, absence of stimulant medication use, and poorer family functioning (14). It is important to note that tic persistence into adulthood does not always mean higher functional impairment, and as previously noted, most adults with TS see an improvement in their functioning over time (17). Although few studies have examined predictors of tic remission, a systematic review of the literature found that lower baseline tic severity in childhood and younger age of TS onset were the only predictors consistently associated with remission across studies (8).

Environmental factors, including psychological stressors, activities of daily living, and specific situations or physical environments are also associated with tic symptom fluctuation. Prior studies have linked transient changes in tic severity to stressful life events, daily stressors, emotional states, fatigue, social attention, and specific settings or activities, including school and public environments (3, 7, 16, 18–22). For example, Barber et al. found that adults with tic disorders retrospectively identified stress and anxiety as common tic triggers across multiple life periods (7). Similarly, Tan et al. found that family-related stress, personal-relationship stress, and school-related stress were associated with higher YGTSS global severity, total tic scores, and impairment scores over time in children with TS or chronic tic disorder (23). Together, these findings suggest that environmental stressors may be relevant to tic expression, particularly in children and adolescents, but their relationship to adult worst-ever tic severity and tic-related impairment remains less clear.

Therefore, the current study aims to expand on the findings of recent literature by (1) examining the relationship between childhood and adulthood tic symptoms and impairment (2), identifying clinical and demographic predictors of adulthood tic symptoms, and (3) assessing the impact of significant life events on adulthood tic symptoms.

## 2 Materials and methods

### 2.1 Participants

Data were collected as part of an ongoing study of TS genetics and phenomenology (1, 24, 25). 758 adults who previously participated in TS genetic studies were sent a follow-up online survey assessing tic symptoms and their impact in adulthood. Of these, 334 individuals (44.1%) responded to the survey, and 292 of 334 respondents (87.4%) completed questions assessing tic severity in both childhood and adulthood and were therefore eligible for inclusion. Participants were excluded if they reported tic onset after age 18, reported childhood worst-ever tics after age 17, adult worst-ever tics before age 18, or adult worst-ever tic age greater than age at follow-up assessment. One participant who reported a childhood worst-ever tic severity score of 0 was also excluded, resulting in a final sample of 258. Participants with intellectual disability, epilepsy, or other genetic or neurological disorders that could confound a TS diagnosis were excluded from the initial studies (24). All study procedures were reviewed and approved by the Mass General Brigham Institutional Review Board.

### 2.2 Measures

#### 2.2.1 Tic severity and impairment

A modified version of the Yale Global Tic Severity Scale (YGTSS) was used to assess tic severity and impairment at three time points (26). First, participants were asked to complete the YGTSS about their current symptoms, defined as “within the last six months.” Next, participants were prompted to think of the time before age 18 when their tics were at their worst and complete the YGTSS for this “childhood worst-ever” tic period. Finally, participants were asked to think of the time when their tics were at their worst as an adult and complete the YGTSS for this “adulthood worst-ever” tic period. 50 participants who reported that they were currently experiencing their worst tics in adulthood were not asked to complete the YGTSS again; instead, their current YGTSS score was also used as their adult worst-ever score. For each time point, YGTSS responses were split into two scores: YGTSS total tic score (YGTSS-TTS) and YGTSS impairment score (YGTSS-Impairment). The two scores were analyzed separately, as each scale is believed to capture a different aspect of TS disease severity (27). The YGTSS-TTS is based on the number, frequency, intensity, complexity, and interference of motor and vocal tics, while the YGTSS Impairment Score captures disruption to or impairment in various aspects of functioning secondary to tics and is thought to be significantly impacted by the presence of co-occurring neuropsychiatric conditions (26–28).

#### 2.2.2 OCD and ADHD

Participants also completed the Tourette Internet-implemented Questionnaire (TIQue) (24, 25), a web-based phenotypic assessment which includes adapted versions of the Florida Obsessive-Compulsive Inventory (FOCI) (29) and the Swanson, Nolan and Pelham (SNAP) Questionnaire (30). The FOCI is a validated self-report instrument that assesses OCD symptoms and severity. Participants were shown a series of OCD symptoms and asked whether they had experienced each symptom ever and/or were experiencing it currently. Then, they were asked a series of questions about their current and worst-ever OCD symptom severity. The SNAP is a parent- and teacher-report instrument that assesses children’s symptoms of attention, hyperactivity, and impulsivity. The SNAP items were administered in a self-report format as part of the TIQue, as previously described (24, 25). Participants were asked to rate how much each of 20 ADHD symptoms described them currently and how much each symptom described them when they were a child. Each question was scored from 0–3, and questions were summed to get a final score. Participants also answered questions about age of symptom onset, childhood impairment, current impairment, and whether they had ever been diagnosed with ADHD.

Lifetime diagnoses of OCD and ADHD were assigned using criteria previously validated by Darrow and colleagues (25). To be assigned a diagnosis of OCD, participants must have endorsed at least four symptoms which either a) Occur for at least one hour per day, with at least mild distress or interference, or b) Occur for less than one hour per day, with at least moderate distress or interference. To be assigned a diagnosis of ADHD, participants must have reached a SNAP score of at least 25 and met the DSM-5 age of onset and impairment in multiple settings criteria.

#### 2.2.3 Clinical and demographic characteristics

Participants were asked to report their age of tic symptom onset, family history of tic disorders (TS and/or motor/vocal tics), and whether they were currently taking medication for their tics. Age at initial interview, age at follow-up assessment, sex, race, and ethnicity were collected as part of the original TS genetic studies (1, 24, 25).

#### 2.2.4 Life events

Participants were first asked to report whether they were experiencing a significant life event at the time of their adulthood worst-ever tics. Those who reported experiencing a significant event were then prompted to describe the event(s) with an open-ended text response. A coding scheme was created to categorize participants’ descriptions of their significant life events into 11 event type categories (work; school; moving; financial; physical health; mental health, psychiatric, & substance use; medication; romantic relationships; friends & family; pregnancy, childbirth, & parenting; and general stress & other) and five emotional valence categories (positive; negative; both positive and negative; neutral; and unknown). Participants’ event descriptions were coded by two independent researchers and could be coded as multiple event type categories, but only one emotional valence category. Discrepancies were discussed until agreement was reached and a final coding was assigned. Interrater agreement for the presence/absence of each event type code was 99.24% before discussion and 100% after. Interrater agreement for the emotional valence category of each participant’s event description was 97.94% before discussion and 100% after.

### 2.3 Statistical analyses

Analyses were conducted using RStudio and Excel. Linear regression was used to examine the relationship between childhood worst-ever and adulthood worst-ever tic symptoms, with age included as a covariate. Pearson correlations were also used to assess unadjusted associations between childhood worst-ever and adulthood worst-ever YGTSS-TTS scores and between childhood worst-ever and adulthood worst-ever YGTSS-Impairment scores. Symptom severity change scores were calculated by subtracting child worst-ever scores from adult worst-ever scores, and the distribution of these change scores was examined. Linear regression was used to model the relationship between each candidate predictor variable (current tic medication status, tic disorder family history, OCD status, ADHD status, sex, and presence/absence of a significant life event) and worst-ever adulthood YGTSS-TTS and YGTSS-Impairment, with age at follow-up assessment included as a covariate. Variables that reached trend level (p < 0.1) were entered into multivariable models, along with worst-ever childhood YGTSS-TTS or YGTSS-Impairment. The frequency of each life event type category and emotional valence category was then examined. Finally, linear regression was used to model the relationship between event emotional valence category and worst-ever adulthood YGTSS-TTS and YGTSS-Impairment scores, with age at follow-up assessment included as a covariate.

## 3 Results

### 3.1 Sample characteristics

The final sample was comprised of 258 adults. Participants’ mean age was 44.3 years (SD = 13.8, range = 26–78). The sample was predominantly male (56.0%), White (93.4%), and non-Hispanic (98.4%). The average age of TS onset was 7.0 years (SD = 2.8, range = 0–16). 95.7% (n = 247) reported having tics at the time of assessment. 66.8% had a family history of tic disorders and 27.9% were taking TS medication at the time of survey completion. 59.6% were assigned a diagnosis of OCD and 26.6% were assigned a diagnosis of ADHD. 59.3% were experiencing a significant life event at the time of their worst-ever adulthood tics. The mean current YGTSS-TTS score was 20.8 (SD = 9.6, range = 0–49) and the mean current YGTSS-Impairment score was 13.9 (SD = 12.2, range = 0–50) (**Figures 1A, D**). Participants reported an average child worst-ever tic age of 12.4 years (SD = 3.1, range = 4–17). At the childhood worst-ever tic time point, the mean YGTSS-TTS score was 29.0 (SD = 9.0, range = 7–50), and the mean YGTSS-Impairment score was 30.4 (SD = 12.2, range = 0–50) (**Figures 1B, E**). Participants reported that their worst-ever adult tics occurred at a mean age of 31.2 years (SD = 12.1, range = 18–68). At the adult worst-ever tic time point, the mean YGTSS-TTS score was 24.4 (SD = 10.0, range = 0–48) and the mean adult worst-ever YGTSS-Impairment score was 21.7 (SD = 14.1, range = 0–50) (**Figures 1C, F**, **Table 1**).

**Figure 1:**
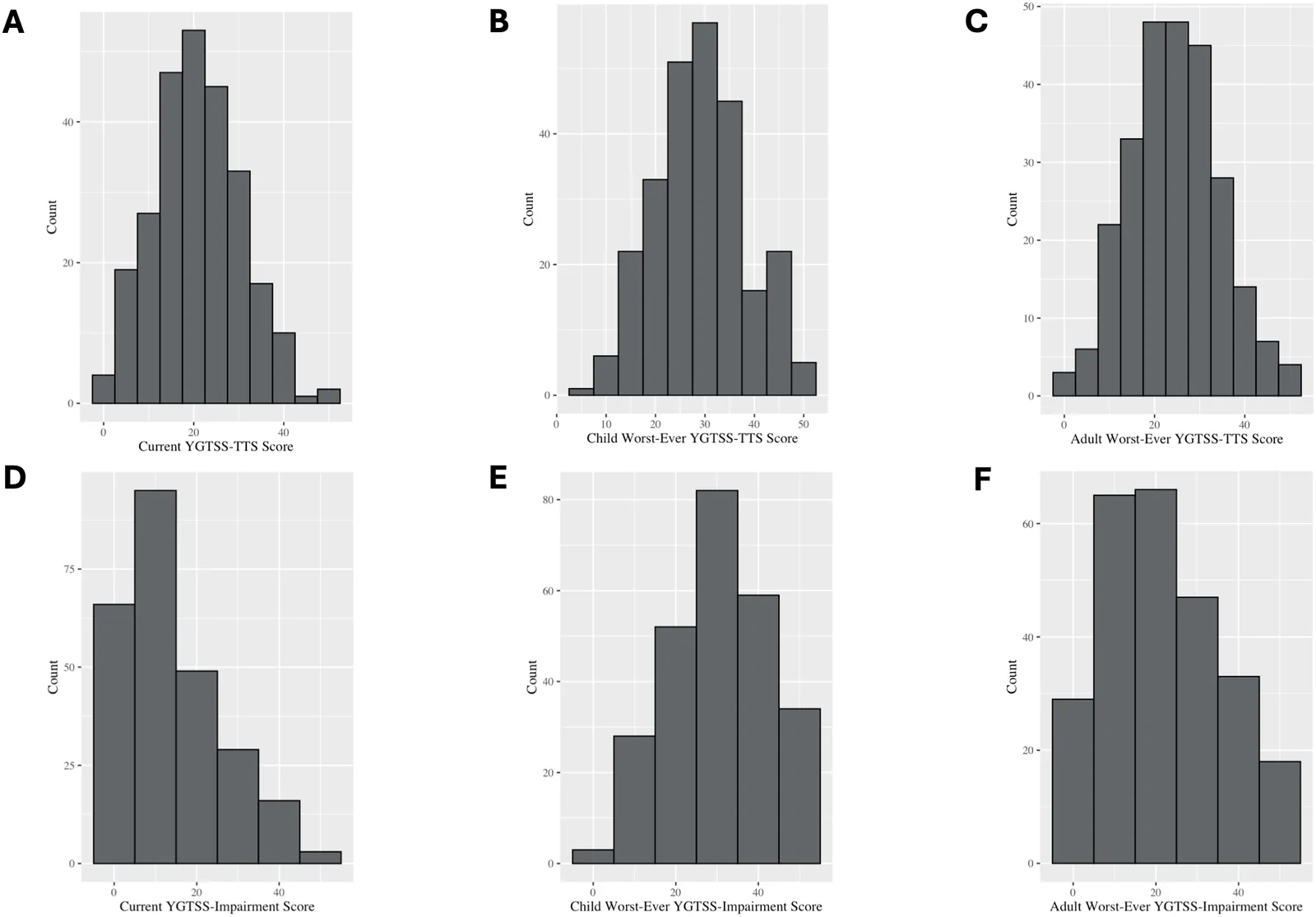
Distributions of current (last 6 months), childhood worst-ever, and adulthood worst-ever YGTSS-TTS **(A–C)** and YGTSS-Impairment **(D–F)** scores.

**Table 1:** Demographic and clinical characteristics of sample population. All numbers calculated excluding missing/unknown values.

Factor	Mean (*SD*, range); *n* (%)
Total number of participants	258
Age	44.3 (13.8, 26–78)
Sex	144 (56.0%) Male
Race	240 (93.4%) White
Ethnicity	252 (98.4%) Non-Hispanic
Age of TS onset, years	7.0 (2.8, 0–16)
Current tic medication status	72 (27.9%) taking medication for tics
Tic disorder family history	155 (66.8%) with family history
OCD status	124 (59.6%) with OCD
ADHD status	67 (26.6%) with ADHD
Significant life event	153 (59.3%) experiencing life event
Current YGTSS-TTS score	20.8 (9.6, 0–49)
Current YGTSS-Impairment score	13.9 (12.2, 0–50)
Age at childhood worst-ever tics, years	12.4 (3.1, 4–17)
Child worst-ever YGTSS-TTS score	29.0 (9.0, 7–50)
Child worst-ever YGTSS-Impairment score	30.4 (12.2, 0–50)
Age at adulthood worst-ever tics, years	31.2 (12.1, 18–68)
Adult worst-ever YGTSS-TTS score	24.4 (10.0, 0–48)
Adult worst-ever YGTSS-Impairment score	21.7 (14.1, 0–50)

### 3.2 Relationship between childhood worst-ever and adulthood worst-ever tic symptoms

A linear regression model including age as a covariate showed that YGTSS-TTS scores during the worst-ever childhood period were significantly associated with YGTSS-TTS scores during the worst-ever adulthood period, β = 0.52, p <.001 (**Table 2**, **Figure 2A**). Similarly, childhood worst-ever YGTSS-Impairment was significantly associated with adulthood worst-ever YGTSS-Impairment, β = 0.45, p <.001, controlling for age at follow-up assessment (**Table 2**, **Figure 2B**).

**Table 2:** Relationship between childhood worst-ever and adulthood worst-ever YGTSS-TTS and YGTSS-Impairment scores, with age as a covariate.

Outcome	Variable	Estimate	Standard error	*t*	*p*
Adult worst-ever YGTSS-TTS	Intercept	4.71	2.86	1.65	0.10
	Child worst-ever YGTSS-TTS	0.52	0.06	8.24	**9.6 × 10^-15^**
	Age	0.10	0.04	2.54	**0.012**
Adult worst-ever YGTSS-Impairment	Intercept	4.97	3.74	1.33	0.19
	Child worst-ever YGTSS-Impairment	0.45	0.07	6.67	**1.6 × 10^-10^**
	Age	0.07	0.06	1.10	0.27

Bold values indicate variables that met significance thresholds of p< 0.05.

**Figure 2:**
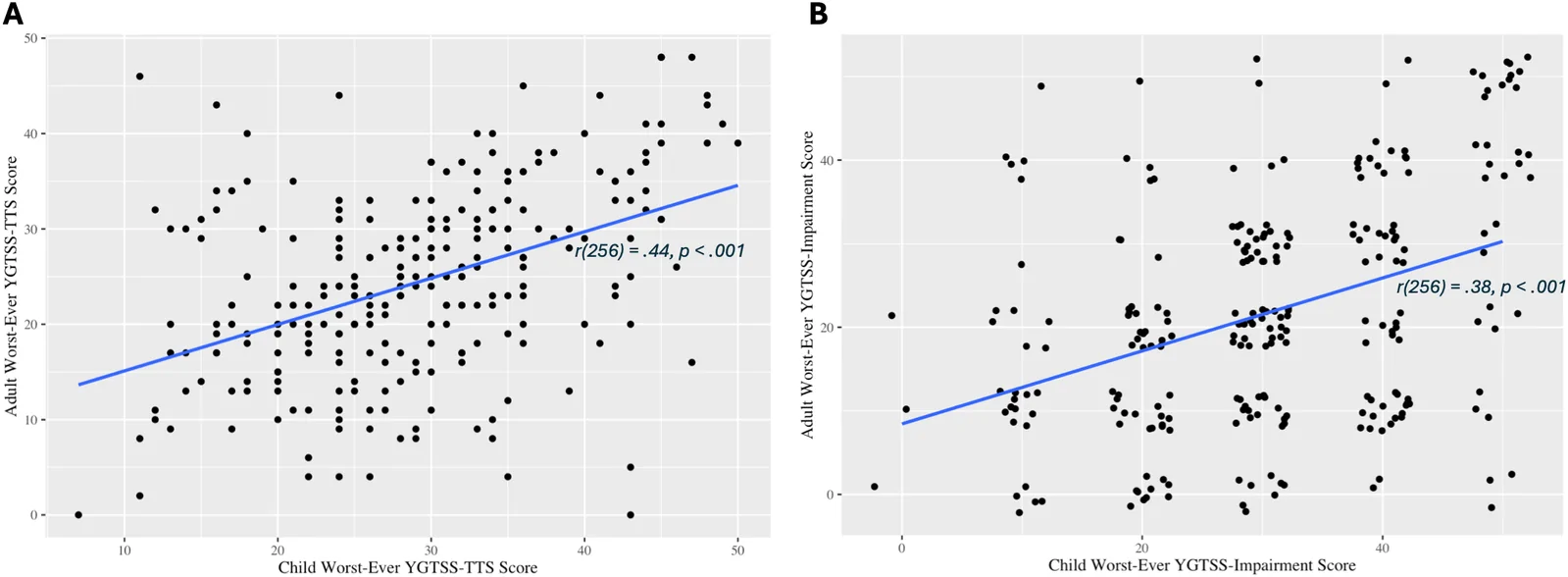
Correlations between childhood worst-ever and adulthood worst-ever YGTSS-TTS **(A)** and YGTSS-Impairment **(B)** scores. Slight random jitter was added to **(B)** for visualization because YGTSS-Impairment scores are measured in 10-point increments.

A YGTSS-TTS change score and YGTSS-Impairment change score were calculated for each participant by subtracting the child worst-ever score from the adult worst-ever score. The mean YGTSS-TTS change score was -4.6 (SD = 10.1, range = -43 to 35), indicating that, on average, tic severity was higher during childhood than adulthood. Similarly, the mean YGTSS-Impairment change score was -8.7 (SD = 14.8, range = -50 to 40), indicating that the average participant reported more severe impairment during their childhood worst-ever tic period than during their adulthood worst-ever tic period (**Figure 3**).

**Figure 3:**
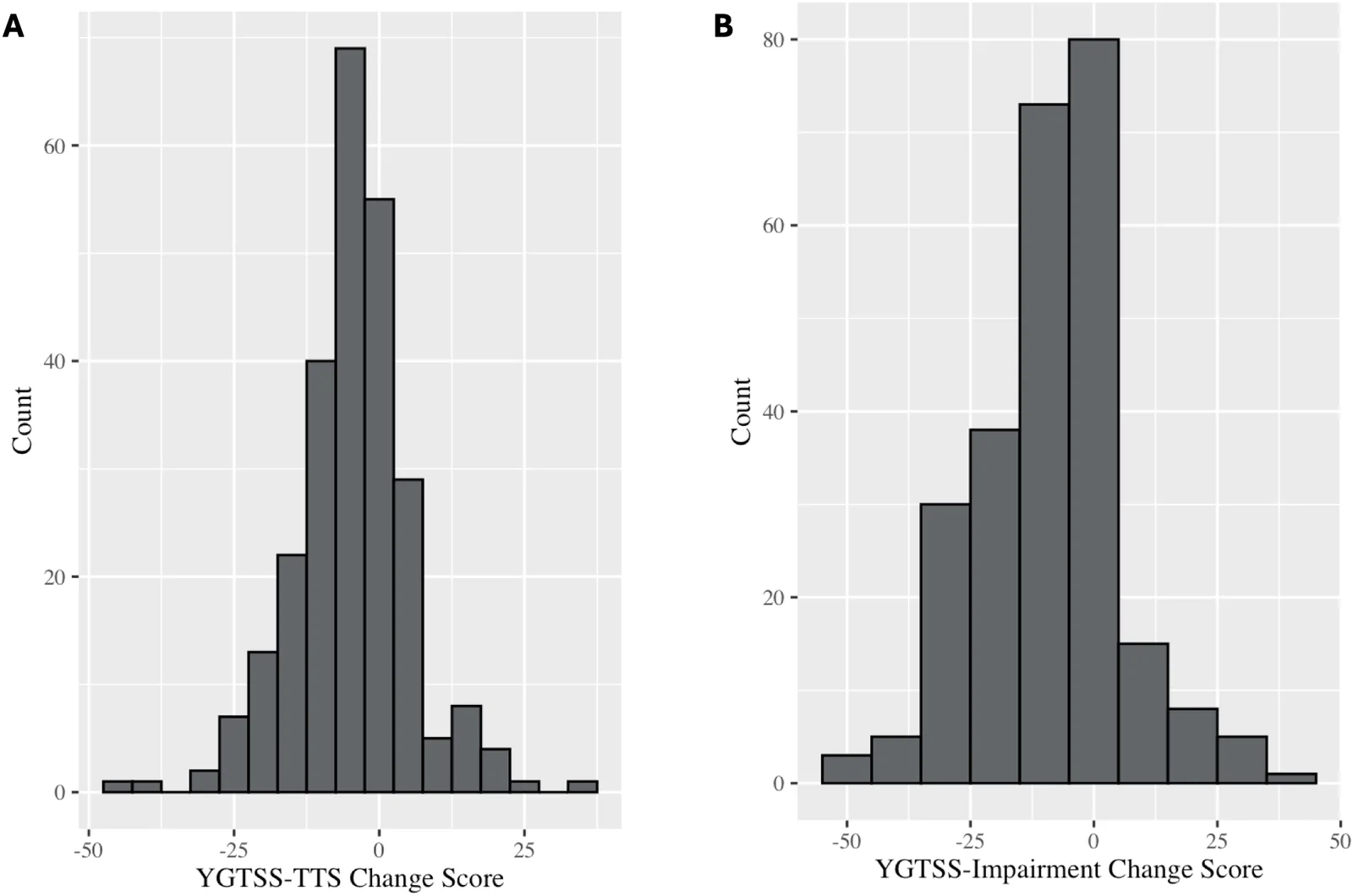
Change in YGTSS-TTS **(A)** and YGTSS-Impairment **(B)** scores from childhood worst-ever to adulthood worst-ever tic periods.

### 3.3 Predictors of adulthood tic severity

To identify predictors of adulthood tic severity, a series of linear regressions was first run to test each candidate predictor’s effect on adult worst-ever YGTSS-TTS score with age as a covariate. Candidate predictor variables included current tic medication status, tic disorder family history, OCD status, ADHD status, sex, and the presence/absence of a significant life event. OCD status (β = 7.26, p <.001), ADHD status (β = 3.73, p = .009), and the presence of a significant life event (β = 3.68, p = .004) were significant predictors of adult worst-ever YGTSS-TTS score. Current tic medication status (β = 2.15, p = .124), tic disorder family history (β = 0.10, p = .942) and sex (β male = -0.95, p = .461) were not significant (**Table 3**). Significant predictors were entered into a multivariable model, along with child worst-ever YGTSS-TTS score, with age as a covariate. OCD status (β = 3.30, p = .018) remained significantly associated with adult worst-ever tic severity. Child worst-ever YGTSS-TTS score was also a significant predictor (β = 0.54, p <.001). ADHD status and the presence of a significant life event were no longer significant in the multivariable model (**Table 4**).

**Table 3:** Relationship between individual participant characteristics, including childhood worst-ever YGTSS scores, and adult worst-ever YGTSS-TTS and YGTSS-Impairment scores.

Outcome	Predictor	Estimate	*p*
Adult worst-ever YGTSS-TTS	Current tic medication status	2.15	0.12
	Tic disorder family history	0.10	0.94
	OCD status	7.26	**3.2 × 10^-7^**
	ADHD status	3.73	**0.0089**
	Sex (male)	-0.95	0.46
	Significant life event	3.68	**0.0037**
	Child worst-ever YGTSS-TTS	0.52	**9.6 × 10^-15^**
Adult worst-ever YGTSS-Impairment	Current tic medication status	6.09	**0.0020**
	Tic disorder family history	0.35	0.86
	OCD status	8.40	**3.8 × 10^-5^**
	ADHD status	2.20	0.28
	Sex (male)	-3.92	**0.030**
	Significant life event	6.85	**1.2 × 10^-4^**
	Child worst-ever YGTSS-Impairment	0.45	**1.6 × 10^-10^**

Each predictor was tested separately for each outcome, with age as a covariate. Female was the reference category for sex.

Bold values indicate variables that met significance thresholds of p< 0.05.

**Table 4:** Predictors of adult worst-ever YGTSS-TTS and YGTSS-Impairment scores using multivariable regression, with age as a covariate.

Outcome	Variable	Estimate	Standard error	*t*	*p*
Adult worst-ever YGTSS-TTS	Intercept	-2.79	3.57	-0.78	0.44
	OCD status	3.30	1.39	2.38	**0.018**
(R^2^ = 0.32	ADHD status	0.33	1.45	0.23	0.82
Adjusted R^2^ = 0.31)	Significant life event	2.15	1.29	1.67	0.098
	Child worst-ever YGTSS-TTS	0.54	0.08	7.09	**2.3 × 10^-11^**
	Age	0.12	0.04	2.80	**0.0056**
Adult worst-ever YGTSS-Impairment	Intercept	-10.11	5.16	-1.96	0.052
	Current tic medication status	4.76	2.03	2.34	**0.020**
(R^2^ = 0.28	OCD status	3.81	1.93	1.98	**0.050**
Adjusted R^2^ = 0.26)	Sex (male)	-4.57	1.80	-2.54	**0.012**
	Significant life event	5.00	1.86	2.69	**0.0078**
	Child worst-ever YGTSS-Impairment	0.41	0.08	5.15	**6.2 × 10^-7^**
	Age	0.13	0.06	1.94	0.054

Predictors were selected from age-adjusted univariable models (p-values < 0.1) and included with the corresponding childhood worst-ever YGTSS score. Female was the reference category for sex.

Bold values indicate variables that met significance thresholds of p< 0.05.

### 3.4 Predictors of adulthood tic impairment

To identify predictors of adulthood tic impairment, the same series of linear regressions was run with adult worst-ever YGTSS-Impairment score as the outcome. Tic medication status (β = 6.09, p = .002), OCD status (β = 8.40, p <.001), sex (β male = -3.92, p = .030), and the presence of a significant life event (β = 6.85, p <.001) were significantly associated with impairment in adulthood. Tic disorder family history (β = 0.35, p = .863) and ADHD status (β = 2.20, p = .277) were not significant (**Table 3**). Significant predictors were entered with child worst-ever YGTSS-Impairment score into a multivariable model, with age as a covariate. Tic medication status (β = 4.76, p = .020), OCD status (β = 3.81, p = .050), sex (β male = -4.57, p = .012), and the presence of a significant life event (β = 5.00, p = .008), as well as child worst-ever YGTSS-Impairment score (β = 0.41, p <.001) were all significant in the multivariable model (**Table 4**).

### 3.5 Significant life events

Of the 258 participants, 153 (59.3%) reported experiencing a significant life event at the time of their worst-ever adulthood tics (**Table 1**). Of those, 122 (79.7%) felt that the event had an effect on their tics, 12 (7.8%) felt it did not have an effect on their tics, and 19 (12.4%) were unsure or preferred not to answer. The event types described by participants varied in frequency (**Figure 4A**). The most common event type was work, with 52 (34.0%) participants including a work-related event in their description, followed by school at 51 (33.3%) and romantic relationships at 43 (28.1%).

**Figure 4:**
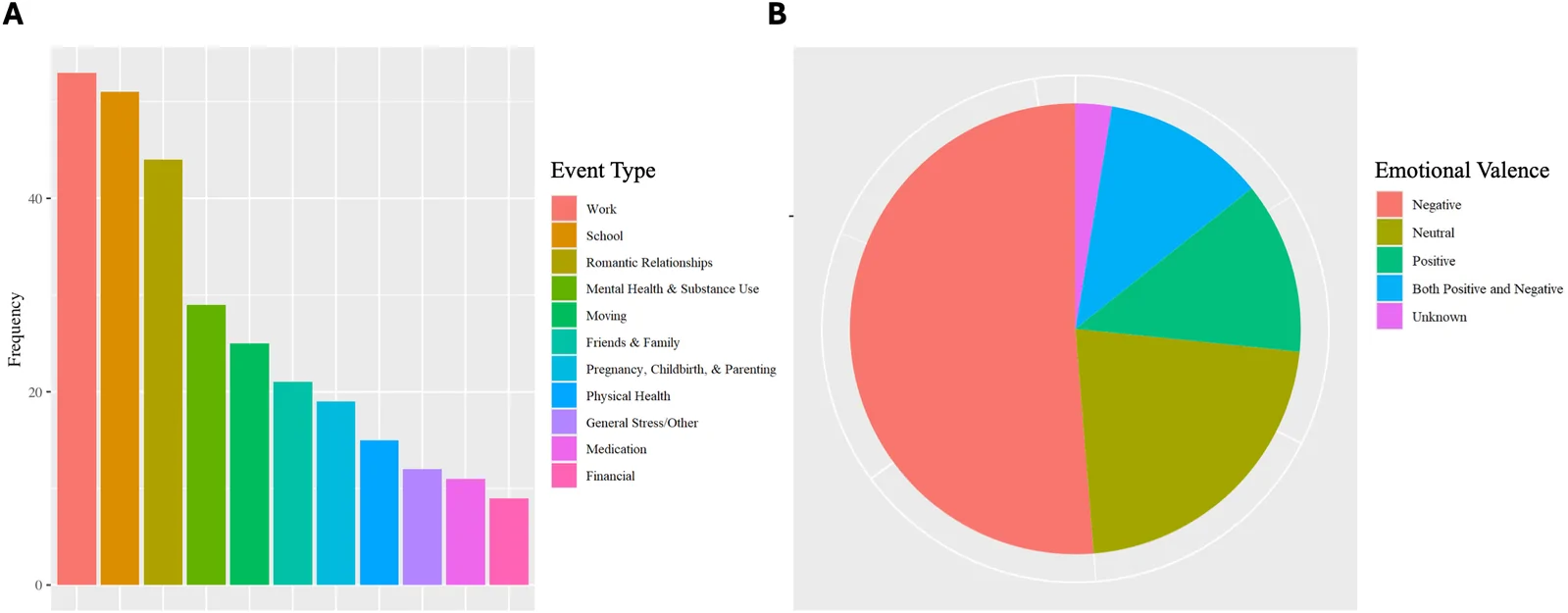
Frequency of coded significant life event types **(A)** and emotional valence categories **(B)** reported during adulthood worst-ever tic periods. Event descriptions could be assigned to multiple event type categories but only one emotional valence category.

Each participant’s response was then categorized according to emotional valence. Of those who reported experiencing a significant life event, 78 (51.0%) described a negative event, 34 (22.2%) described a neutral event, 19 (12.4%) described a positive event, 18 (11.8%) described an event that was both positive and negative, and 4 (2.6%) did not describe their event or described an event with unclear emotional valence (**Figure 4B**). There was a significant association between negative and neutral life events and adult worst-ever YGTSS-TTS and YGTSS-Impairment scores, in linear regression analyses, compared to those not reporting a significant life event at the time of adulthood worst-ever tics (**Table 5**). Positive, both positive and negative, and unknown emotional valence events were not significantly associated with tic severity or impairment.

**Table 5:** Relationship between significant life event emotional valence and adult worst-ever YGTSS-TTS scores and YGTSS-Impairment scores, with “no life event reported” as the reference category and age as a covariate.

Outcome	Variable	Estimate	Standard error	*t*	*p*
Adult worst-ever YGTSS-TTS	Intercept	20.14	2.25	8.97	**<2.0 × 10^-16^**
	Event valence (Positive)	1.80	2.47	0.73	0.47
(R^2^ = 0.043	Event valence (Negative)	3.59	1.48	2.42	**0.016**
Adjusted R^2^ = 0.020)	Event valence (Neutral)	5.30	1.96	2.71	**0.0072**
	Event valence (Both Positive & Negative)	2.57	2.54	1.01	0.31
	Event valence (Unknown)	5.46	5.04	1.08	0.28
	Age	0.05	0.05	1.03	0.31
Adult worst-ever YGTSS-Impairment	Intercept	16.99	3.11	5.46	**1.1 × 10^-7^**
	Event valence (Positive)	-1.31	3.42	-0.38	0.70
(R^2^ = 0.086	Event valence (Negative)	8.43	2.05	4.11	**5.4 × 10^-5^**
Adjusted R^2^ = 0.064)	Event valence (Neutral)	7.72	2.71	2.85	**0.0048**
	Event valence (Both Positive & Negative)	6.38	3.52	1.81	0.071
	Event valence (Unknown)	9.95	6.99	1.43	0.16
	Age	0.01	0.06	0.22	0.83

Bold values indicate variables that met significance thresholds of p< 0.05.

## 4 Discussion

In this sample of 258 adults with TS, participants on average reported more severe and more impairing tics during childhood than during adulthood. This supports a pattern of tic improvement over time, and is consistent with previous studies (6, 9). On average, participants reported an almost 5-point improvement in YGTSS-TTS scores between the child and adult worst-ever tic periods, corresponding to a 16% reduction in severity, and an almost 9-point improvement in YGTSS-Impairment scores, corresponding to a 28.6% reduction in tic-related impairment. The larger change in child and adult impairment scores compared to severity scores is consistent with prior long-term outcome studies showing that tics often persist into adulthood even when day-to-day impairment is reduced, and may indicate that individuals with TS learn to adapt to their tics even when they don’t experience true symptom reduction (8–10). However, this observed difference between impairment and severity scores may also in part be due to differences in scoring resolution between the two YGTSS subscales. Although both scales range from 0 to 50, the YGTSS-TTS is scored in 1-point increments, whereas the YGTSS-Impairment score is assigned in 10-point increments, making direct comparison of change magnitude across the two subscales difficult.

In addition to replicating previous reports of tic improvement in adulthood, our results also show a strong association between child and adult worst-ever tic severity and impairment, with childhood severity and impairment emerging as the strongest predictors of corresponding outcomes in adulthood. These findings support previous observations that childhood worst-ever tic symptom severity is an important predictor of adult tic severity (13, 14).

In addition to severity of childhood tic symptoms, several additional predictors of adulthood tic severity and impairment were identified. First, tic medication use was associated with increased tic impairment (but not tic severity) in adulthood. This likely reflects the fact that individuals with significant impairment are more motivated to pursue treatment for their tics, including pharmacological treatments. In addition, we found that co-occurring OCD, but not co-occurring ADHD, in childhood was associated with increased tic severity and impairment in adulthood. This is partially aligned with previous findings – for example, Mataix-Cols and colleagues found that the presence of tics in adulthood was predicted by psychiatric comorbidities in childhood, including ADHD and OCD (15). Finally, female sex was associated with greater tic-related impairment, but not tic severity, during the worst-ever adulthood tic period. This is partially consistent with Ricketts et al., who found that female sex was associated with greater tic severity and tic-related impairment in older adolescents and adults and with a smaller reduction in tic-related impairment at 11-year follow-up (14).

This study also explored how significant life events may affect adulthood tic symptoms. Over half of the sample reported experiencing a significant life event at the time of their worst-ever adulthood tics. As expected, those who reported a significant life event also tended to have higher rates of impairment than those who did not report such a life event. These findings are consistent with Horesh and colleagues (2018), who found that major life events correlated with more severe symptoms among children with TS (3), and align with Barber et al., who found that adults with tic disorders retrospectively identified stress and anxiety as common tic triggers across multiple life periods (7). The mechanism by which significant life events affect tics is unknown. Stressful events may induce an allostatic response in the brain, disrupting homeostatic balance and triggering tic exacerbation (31). Further, research has shown that anxiety can negatively impact symptoms in a subset of tic patients (32).

To further investigate the effect of significant life events, participants’ descriptions of their life events were examined. Each description was first coded by emotional valence. Compared to participants with no reported life event, participants who described negative or neutral life events had higher tic severity and impairment, whereas positive life events were not significantly associated with either outcome. These findings support the hypothesis that stress and anxiety associated with significant life events may act on neurobiological mechanisms that exacerbate tic symptoms. While this result could be due to the small number of participants who reported positive life events at the time of their adulthood worst-ever tics, the lack of participants who experienced positive events at this time is itself an indicator that positive events may be less frequently associated with severe tic symptoms. The type of life event was then assessed in relation to tic severity. The most frequent events described were related to work, school, and romantic relationships. As these events have the potential to be highly stressful, this finding aligns with previous work demonstrating that tic disorder patients consistently report stress and anxiety as triggers for increased tic symptoms (7).

This study has several notable limitations. First, data relied on retrospective reporting, leaving room for recall bias or unintentional over-weighting of more recent symptoms compared to more distant symptoms (recency bias). Participants also provided self-report data—tic severity and impairment were not directly assessed by a clinician. Second, limited data was collected for several variables, especially significant life events. Participants were not directly asked to label their events as positive or negative or to assign them to categories; instead, these labels were retroactively assigned by the research team. Therefore, it is not possible to confirm whether participants’ own perspectives on these events would align with the study team’s coding. Third, we did not collect information on premonitory urges or other factors (e.g., genetic risk) that may have been associated with tic persistence. Fourth, the sample did not include individuals with diagnoses that were previously believed to “confound” a TS diagnosis, such as intellectual disability or autism spectrum disorder (ASD). Fifth, participants were largely white and non-Hispanic, making it difficult to generalize these results to the entire diverse tic disorder community.

Future research should investigate these factors longitudinally with a diverse sample using a mix of detailed questionnaires that ask participants to rate the significance of the measured life events, as well as including observer ratings of tic symptoms. Furthermore, although the YGTSS-Impairment score captures overall disruption, future studies should refine this measurement by assessing specific domains of impairment, including family functioning, peer functioning, intimate relationship functioning, and academic or work achievement. In addition, there are many other potential predictors of adulthood tic persistence that could be investigated, and a larger sample size may reveal associations between variables that this study was unable to identify. Finally, combining these phenotypic factors with genetic data (such as PRS scores) could further the ability to identify specific, personalized tic trajectory predictions.

## Data Availability

The raw data supporting the conclusions of this article will be made available by the authors as allowed by the Mass General Brigham Institutional Review Board policies.
